# SARS-CoV-2 infection in patients with inborn errors of immunity due to DNA repair defects: DNA repair deficiency and COVID19

**DOI:** 10.3724/abbs.2022071

**Published:** 2022-06-15

**Authors:** Yating Wang, Hassan Abolhassani, Lennart Hammarström, Qiang Pan-Hammarström

**Affiliations:** Department of Biosciences and Nutrition Karolinska Institutet SE-14183 Huddinge Sweden

**Keywords:** COVID-19, DNA repair mechanism, inborn errors of immunity, primary immunodeficiency

## Abstract

Clinical information on severe acute respiratory syndrome coronavirus 2 (SARS-CoV-2) infection in patients with inborn errors of immunity (IEI) during the current Coronavirus disease 2019 (COVID-19) pandemic is still limited. Proper DNA repair machinery is required for the development of the adaptive immune system, which provides specific and long-term protection against SARS-CoV-2. This review highlights the impact of SARS-CoV-2 infections on IEI patients with DNA repair disorders and summarizes susceptibility risk factors, pathogenic mechanisms, clinical manifestations and management strategies of COVID-19 in this special patient population.

## Introduction

Coronaviruses (CoVs) belong to the subfamily Coronaviruinae, in the entity of the Nidovirales. Severe acute respiratory syndrome CoV-2 (SARS-CoV-2) is an enveloped, single positive-strand RNA (ssRNA) virus. SARS-CoV-2 can infect mammals and lead to lethal human disease [1]. It consists of several structural proteins, including the spike (S), envelope (E), membrane (M) and nucleocapsid (N) [2]. SARS-CoV-2 enters host cells through three routes: receptor-dependent cell membrane fusion, receptor-dependent endocytosis, or antibody-dependent endocytosis [3]. Angiotensin-converting enzyme II (ACE2) and transmembrane serine protease 2 (TMPRSS2) are the main receptors for SARS-CoV-2, mediating internalization through specific interaction with the S protein. The viral genomic RNA can be translated as two large open reading frames (ORF) after fusion at the membrane, ORF1a and ORF1b. Many individual non-structural proteins (NSPs) are subsequently expressed including viral replication and transcription complex proteins. Accordingly, double-membrane vesicles, convoluted membranes and tiny open double-membrane spherules can be translated and provide a viral genomic RNA replication microenvironment. Viral structural products are transferred into the endoplasmic reticulum (ER) and subsequently into the ER-Golgi intermediate compartment (ERGIC). Thereafter, newly-synthesized viral genomic RNA is encapsulated and secreted from the host cell by exocytosis [4].

Except for SARS-CoV-2, influenza virus and respiratory syncytial virus (RSV) are the main pathogens associated with severe viral respiratory disease worldwide [5]. Severe symptoms of respiratory viral infectious diseases are usually observed only in a minority of infected individuals and genetic predisposing factors affecting innate and adaptive immune responses have been described in subgroups of patients [6].

## Innate and Adaptive Immune Response Against SARS-CoV-2

**Figure 1:**
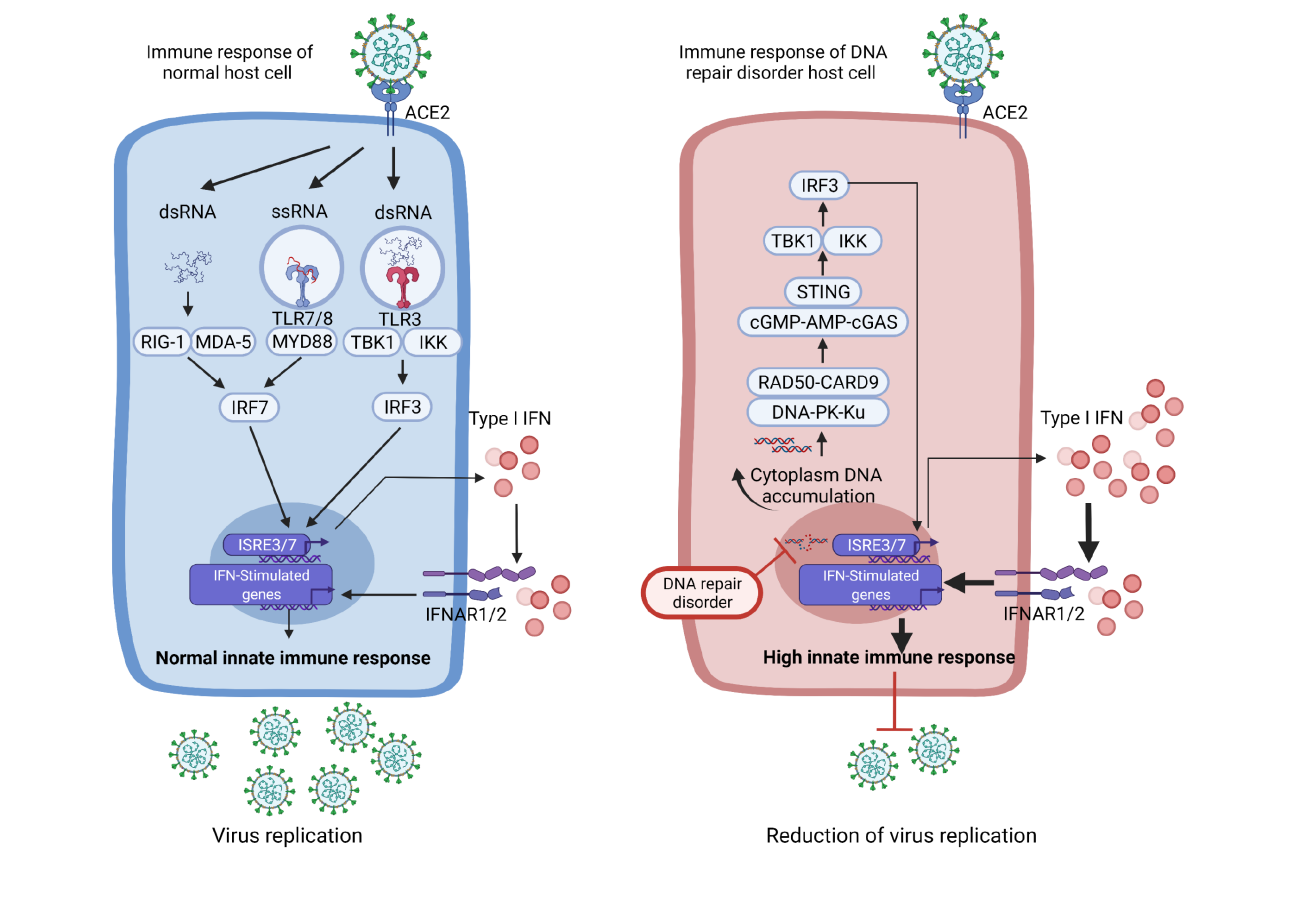
Figure 1 Comparison of the innate immune response to SARS-CoV-2 between normal host cells and DNA repair protein defective host cells In normal host cells (left), following infection of SARS-CoV-2, RIG-1-MDA-5 senses cytosolic dsRNA, TLR7/8/3 sense ssRNA and dsRNA, respectively. Subsequently MYD88 and TBK1-IKK activate IRF3/IRF7 and regulate ISRE7/8 expression, leading to the expression of type I IFN. Type I IFN then primes an antiviral programme in both infected cells and neighboring cells through IFNAR1/2 by regulating the expression of IFN-stimulated genes. In DNA repair protein defective host cells (right), before SARS-CoV-2 infection, unrepaired DNA damages induce cytoplasmic DNA accumulation. Following the recognition by the RAD50-CARD9 and DNA-PK-Ku complexes, the type I IFN system is primed via the STING pathway. Overactivation of type I IFN promotes antiviral activity and reduces virus replication. IRF3: transcription factors interferon (IFN)-regulatory factor 3; TLR: Toll-like receptors (TLRs); IFNAR1/2: interferon-α receptor; TBK1: TANK-binding kinase 1; IKK: IkappaB kinase; DNA-PK: DNA-dependent protein kinase; Ku: Ku70-Ku80 heterodimer; RAD50: DNA repair protein RAD50; CARD9: Caspase recruitment domain family member 9; cGMP-AMP: cyclic guanosine monophosphate-adenosine monophosphate; STING: Stimulator of interferon genes.

In recent studies, innate immunity has shown its importance to combat SARS-CoV-2 infection. The antiviral innate immune response against SARS-CoV-2 can be initiated by endosomal RNA receptor recognition when Toll-like receptor 7/8 (TLR7 and TLR8) recognize SARS-CoV-2 endocytosed single-strand RNA (ssRNA). Double strand RNA (dsRNA) of the virus in the cytoplasm can be sensed by the cytosolic RNA sensor, RIG-1/MDA-5 (Retinoid-inducible gene/ melanoma differentiation-associated gene 5). Subsequently, the adaptor proteins, myeloid differentiation factor 88 (MYD88) and mitochondrial antiviral signaling protein (MAVS), are recruited, triggering and amplifying downstream signaling pathways through activation of several transcription factors, such as nuclear factor kappa B (NF-κB) and interferon response factor 3/7 (IRF3/7) [7]. It results in the production of inflammatory molecules, including interleukin 6 (IL-6), tumor necrosis factor (TNF) as well as interferons (IFNs) [1]. Among these factors, IFNs are especially critical for the immune response against SARS-CoV-2 ( Figure 1). Accordingly, genetic defects in type I and type III IFN regulatory genes are likely to underlie severe forms of COVID-19 in young adults [8].

**Figure 2:**
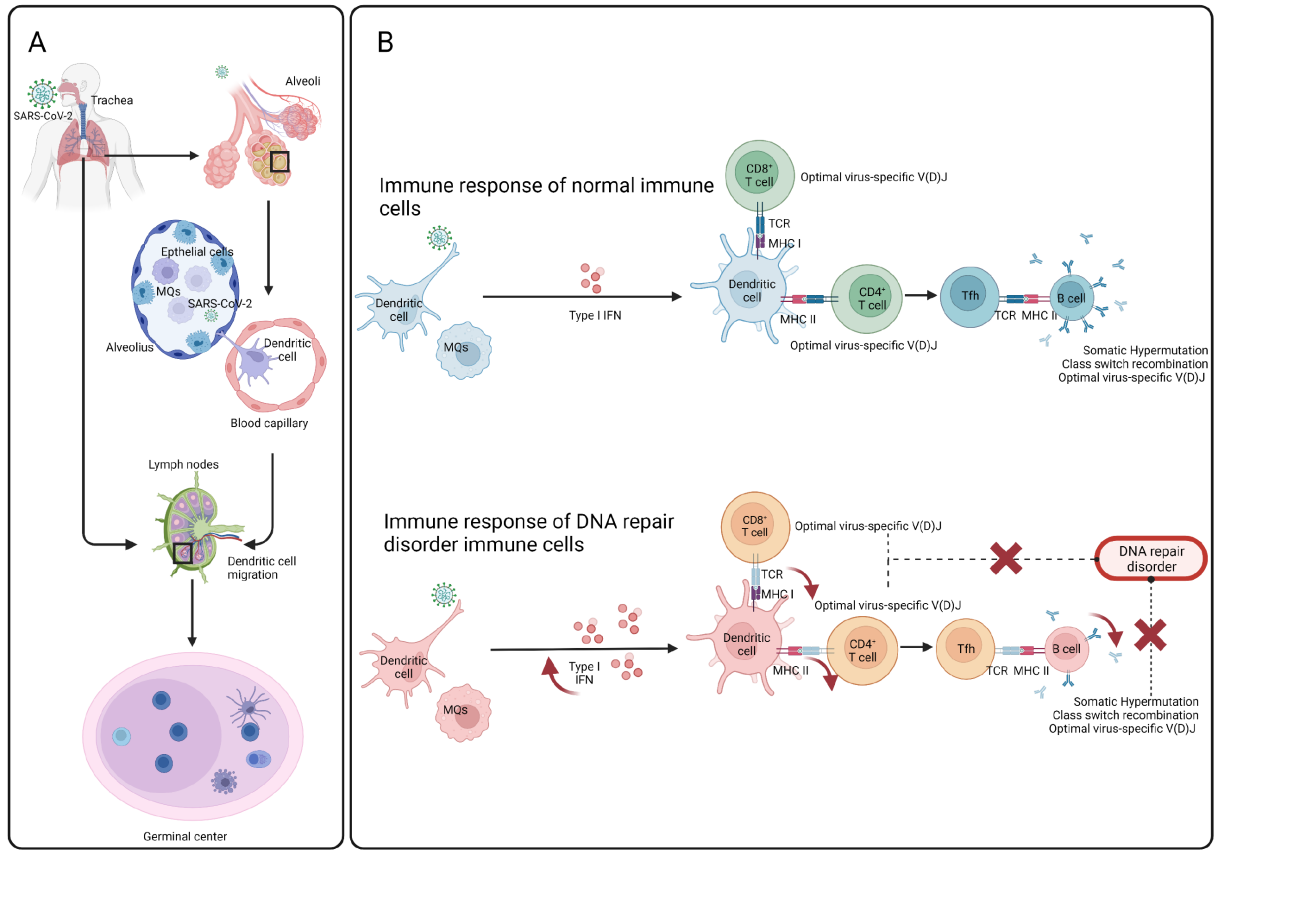
Figure 2 Comparison of the adaptive immune responses to SARS-CoV-2 between normal host cells and DNA repair protein defective host cells (A) The process of SARS-CoV-2 priming of the adaptive immune response in the lung is depicted. (B) The comparison of adaptive immune responses in normal immune cells and DNA repair protein-deficient cells is highlighted. Tfh: T follicular helper cells; TCR: T cell receptor; MHC: major histocompatibility complex.

The central antigen-presenting cells in viral infections, plasmacytoid dendritic cells (pDCs), are responsible for activating antigen-specific adaptive immune cells in the respiratory tract, thus linking the innate and adaptive immune systems. Therefore, genetic defects in essential functional genes of pDCs have also been identified in patients with severe respiratory infections, including SARS-CoV-2 [9]. Effective foreign antigen recognition requires receptors, which can sense unique antigen/major histocompatibility complex (Ag/MHC) combinations. Adaptive immunity is likely to be of critical importance for protecting individuals against SARS-CoV-2 by viral clearance and the persistence of antiviral immunity. In addition to B cell response, which results in the production of virus-specific binding and neutralizing antibodies as well as long-term protection through B cell memories, T cells have also been shown to play an important role in the adaptive immune response against SARS-CoV-2. CD4 ^+^ T cells function by stimulating B cells, helping/activating CD8 ^+^ T cells, and recruiting more innate immune cells to the infectious sites [10]. IFNγ , as the main cytokine produced by Th1 cells which are differentiated from CD4 ^+^ T cells, is crucial for the development of protective immunity against severe viral infections [11]. CD8 ^+^ T cells are responsible for the clearance of SARS-CoV-2-infected cells, and a virus-specific CD8 ^+^ T cell response is associated with milder COVID-19 symptoms [12]. COVID-19 patients with severe/critical disease display higher numbers of macrophages and neutrophils, and reduced proportions of pDCs and T cells than patients with moderate infection [13] ( Figure 2). It suggests that an insufficient T cell response increases the severity of SARS-CoV-2 infection.

B cells respond to virus infection during the second week after the symptom onset in COVID-19 patients, and produce antibodies against various structural proteins [14]. The antibodies are initially of the IgM class, followed by IgG and IgA after 8–24 days [15], through a process named isotype switching (also known as immunoglobulin class switching). Specific IgG1 and IgG3 against SARS-CoV-2 are the IgG subclasses preferentially expressed in serum [16]. Specific IgA contributes to virus neutralization to a greater extent compared to IgG during the early phase of infection, but the serum concentration decreases one month after the infection, though the neutralizing IgA can be detectable in saliva for a longer time [17]. The IgG response and neutralizing titers decrease gradually overtime but stabilize at a low level after 6 months [ 18– 20] . The virus-specific memory B and T cell responses, however, persist in the majority of the convalescent donors tested for up to 15 months [19].

## DNA Damage Response and Repair

DNA damage occurs frequently in human cells due to exposure to physiological and pathological events, including oxygen radicals, ionizing radiation or during DNA replication, genomic rearrangements, meiosis and adaptive immune development [ 21, 22] . Single-strand breaks (SSBs) are the most common type of DNA damage, while double-strand breaks (DSBs) are more harmful and may even be lethal to the cells [ 23, 24] . A DNA damage response (DDR) is initiated by the induction and detection of the DNA damage. Various forms of DNA damage activate specific signaling and DNA repair pathways, and more than 400 proteins have been suggested to be involved in these processes [25]. Six core DNA repair pathways have been identified depending on different types of DNA lesions, including base excision repair (BER), mismatch repair (MMR), nucleotide excision repair (NER), homologous recombination (HR), non-homologous end joining (NHEJ), and interstrand cross-link repair (ICL) [ 26, 27] . BER and MMR are responsible for single nucleotide base modifications and small insertions/deletions, respectively, whereas NER is used for repairing bulky DNA lesions. The ICL pathway repairs the damage when two DNA strands are covalently bound by error. HR and NHEJ are responsible for DSB repair.

The MRN complex, MRE11 (meiotic recombination 11)-RAD50-NBS1 (Nijmegen breakage syndrome 1), is a crucial factor involved in both the HR and NHEJ pathways [ 28– 32] . The MRN complex binds to sister chromatids with DSB sites and actives Ataxia telangiectasia mutated (ATM), a central element of DDR, for further repair [33]. HR is a complex pathway that uses the sister chromatid as the template to repair the DSBs. The C-terminal binding protein–interacting protein (CtIP) is likely to stimulate the exonuclease activity of the MRN complex [34]. To coat replication protein A (RPA) on single-strand DNA (ssDNA) for later repair process, extensive ssDNA is degraded by the EXO1 (Exonuclease 1)-BLM (Bloom syndrome protein)-DNA2 (DNA replication helicase 2) complex [35]. With the assistance of a sister chromatid searching (which needs homologous length >100 bp, mainly available during the S and G2 phases of the cell cycle) by BRCA1-PALB2-BRCA2, DNA polymerase synthesizes the DNA strand and BLM resolves the Holliday junction produced by the homologous replication [36]. Finally, DNA ligase I/III (LIG1/3) re-joins the fragments of DNA breaks [37]. Moreover, single-strand annealing (SSA) can resect the breakage end with the assistance of >50 bp overlapping homology of the sequence instead of using a sister chromatid template in the absence of D-loop components as an alternative repair for HR during the S and G2 phases. On the other hand, NHEJ is used when there is no guidance template, particularly during the G0 and G1 phases of the cell cycle. Classical NHEJ (C-NHEJ) performs repairs by using minimal complementary base pairs (1–4 nucleotides) [ 38– 41] . Ku70-Ku80 recruits the DNA-dependent protein kinase catalytic subunit (DNA-PKcs or PRKDC) to initiate the NHEJ process. Other key DNA repair proteins involved in the C-NHEJ process are Artemis (DCLRE1C), DNA ligase IV (LIG4), X-ray repair cross-complementing protein 4 (XRCC4), and XRCC4-like factor (XLF) [ 42– 46] . Alternative NHEJ (A-EJ) repair of DSBs utilizes 2–20 bp overlapping bases and recruits CtIP, before DNA polymerase theta (Polθ), and poly ADP-ribose polymerase I (PARP1) to anneal the cleavages [ 47– 50] . XPF-ERCC1-complex cuts 3’-flap ssDNA before LIG1/III ligation and may also play a role in A-EJ [ 51– 53] .

The critical roles of ATM in cell cycle regulation and DDR have been described previously [54]. Together with the MRN complex or BLM, ATM takes part in HR by the regulation of short and long DNA end resection, respectively. To complete the HR function of ATM, RAD51 gets involved in various replication fork processes and strand pairing stages. During NHEJ, ATM regulates DNA-PKs activity. Furthermore, TP53-binding protein (53BP1) can be phosphorylated by ATM and subsequently regulates NHEJ by recruiting RAP1-interacting factor 1 homolog (RIF1) for shielding and PTIP for end-joining. Moreover, ATM regulates XRCC1, which is recruited by PARP1 through checkpoint kinase 2 (CHK2) during the BER process [55].

DNA repair defects in human have been described to predispose to growth retardation, genomic instability [56], cytoplasmic DNA accumulation [57], cell apoptosis [58], radiosensitivity [59], immunodeficiency [60], and cancer [61].

## The Relationship of DNA Damage Response and Immune Response Against SARS-CoV-2

As mentioned above, type I IFN immunity is critical for the innate immune response against SARS-CoV-2. The type I IFN pathway can be induced by viral pathogens recognized by members of the TLRs family. TLRs localized in endosomes can recognize dsRNA (TLR3), ssRNA (TLR7/8), and CpG DNA (TLR9), respectively [ 62– 64] . The type I IFN pathway can also be activated by the cyclic guanosine monophosphate (GMP)–adenosine monophosphate (AMP) synthase (cGAS)-stimulator of IFN genes (STING) pathway [ 65– 67] . cGAS is a sensor that can detect both cytosolic self- and foreign-oligonucleotide [68]. Activation leads to the production of second messenger cyclic GMP-AMP (cGAMP) which can bind to the adaptor protein STING and activate the type I IFN pathway by activation of TANK-binding kinase 1 (TBK1) and inhibitor of NF-κB kinases (IKK) [68]. Dysfunction of ATM results in the accumulation of micronuclei in the cytosol which contains self-DNA [ 65, 67, 69] . The DNA-PKcs-KU70/80 complex, which is required by the NHEJ process, can also sense DNA in the cytoplasm [ 70, 71] . The double-strand DNA (dsDNA)-RAD50-CARD9 complex, as part of DSBs sensor, can also detect the cytoplasmic dsDNA [72]. Subsequently, the type I IFN system will be activated through the cGAS-cGAMP-STING pathway [65]. In summary, patients with DNA repair disorders may have a higher degree of accumulation of cytoplasmic self-DNA, which presumably can stimulate the STING pathway and lead to a higher type I IFN mediated innate immune response against SARS-CoV-2 infection ( Figure 1).

DNA repair processes may also play a critical role in the adaptive immunity against SARS-CoV-2, where three main immunological diversification mechanisms require efficient and regulated DNA repair function, T- and B-cell receptor generation (V(D)J recombination), immunoglobulin class-switch recombination (CSR) and somatic hypermutation (SHM) [22] ( Figure 2). The V(D)J recombination occurs during early B and T cells development, which is initiated by the recombinase-activation gene (RAG) protein-induced DSBs, requires the C-NHEJ machinery. Thus, insufficient DNA repair may result in a lack of T and B cell receptor generation, which leads to a decrease in the number of functional T- and B cells required for immunity against viral infections like SARS-CoV-2 [73].

Both CSR and SHM occur in the germinal center during late B cell development. CSR is initiated by activation-induced cytidine deaminase (AID) through cytosine (C) to uracil (U) deamination [ 74, 75] . The AID-induced lesions engage the activity of either the uracil DNA glycosylase (UNG)-mediated BER or the MSH-mediated MMR pathway, creating nicks or DSBs in the variable or switch regions to initiate SHM or CSR, respectively [76]. Additional factors, such as DNA repair proteins, EXO1 and DNA polymerase η (POLH) have also been implicated in the SHM process [ 77, 78] . Although IgG and IgA switching occur frequently during the SARS-CoV-2 infection, no or lower level of SHM was observed in the early stage of infection [ 15, 79, 80] . The mean rate of SHM of 774 published SARS-CoV-2 targeted antibodies (2.64%) is slightly lower than the SHM rate in specific antibodies in other acute infections, such as SARS-CoV-1, Ebola and Zika viruses, where the SHM rate is 3.42% to 7.46% [15]. However, in the late stages of infection (> 6.2 months after infection), the SHM activity after SARS-CoV-2 is normalized in memory B cells and may contribute to the enhanced neutralizing breadth against the virus [ 81, 82] . Thus, DNA repair proteins are essential for the development of adaptive cellular and humoral immunity during viral infections. As a result, DNA repair disorders are expected to have a negative association with adaptive immune responses against SARS-CoV-2.

## Association of Viral Infection and Disease Pathology in Inborn Errors of Immunity

Inborn errors of immunity (IEIs) are a group of diseases characterized by an increased susceptibility to infections and immune dysregulation due to genetic defects affecting the development or/and function of the immune system. There are over 450 IEI-related genes classified into ten categories based on the International Union of Immunological Societies (IUIS) classification [83]. IEI patients have many direct or indirect defects in antiviral pathway components, thus presenting a higher susceptibility to viral infections [84]. Some monogenic IEIs are well known to have susceptibility to specific viral infections. Severe herpes simplex virus (HSV) is associated with TLR3 pathway deficiencies (including *TLR3*, *UNC93B1*, *TRIF*, *TRAF3*, and *TBK1*) [ 85, 86] . Whereas severe Epstein-Barr virus (EBV)-related diseases are mainly due to defects in activation and function of cytotoxic CD8 ^+^ T cells ( *SH2D1A*, *MAGT1*, *ITK*, *CD27*, and *CD70*) [ 87– 91] , Furthermore, severe human rhinoviruses infections can be seen in RNA sensing gene ( *IFIH1*, encoding MDA5) deficiency [ 92– 94] . Other reported monogenic IEI defects with severe viral susceptibility include influenza virus infection ( *IRF7*, *IRF9*, *STAT1*, and *STAT2*) [ 95, 96] , varicella-zoster virus (VZV) pneumonia ( *MCM4*, *CTPS1*, and *FCGR3A*) [97], human papillomavirus severe warts ( *GATA2*, *TMC6*, *TMC8*, *CIB1*, and *CXCR4*) [98], side-effects of vaccination with attenuated viruses ( *IFNAR1*, *IFNAR2*, *STAT2*, and *IRF9*) and more recently, life-threatening SARS-CoV-2 infection ( *TLR3*, *TLR7*, *IFNAR1*, *IFNAR2*, *IRF7*, *IRF9*, *TICAM1*, *TBK1* and *IRF3*) [ 9, 99– 102] . Of note, MYD88-deficient patients have previously been reported to suffer from severe invasive bacterial infections rather than viral infections [103]. However, a study of SARS-CoV-1 has demonstrated a vital role of MYD88 in innate immune signaling and inflammatory cell recruitment [104]. Moreover, three MYD88-deficient patients have recently been reported to suffer from severe COVID-19 and required intensive care unit (ICU) admission during the recent SARS-CoV-2 pandemic [105].

Among the known IEI, 21 autosomal recessive DNA-repair disorders have been identified. DNA repair-associated genes include *RAG1*/ *2*, *DCLRE1C*, *PRKDC*, *NHEJ1*, *LIG4*, *ATM*, *NBS1*, *BLM*, *DNMT3B*, *ZBTB24*, *CDCA7*, *HELLS*, *PMS2*, *RNF168*, *MCM4*, *OLE1*/ *2*, *NSMCE3*, *ERCC6L2* and *GINS1* [83]. *RAG1*/ *2*, *DCLRE1C*, *PRKDC*, *NHEJ1*, and *LIG4* result in isolated non-syndromic combined immunodeficiency (CID, due to low-T low-B CID) while defects in other genes cause syndromic combined immunodeficiencies (due to the additional roles of these genes in non-immune cells) [ 83, 106] . These DNA repair disorders are reported equally in both genders and can underlie susceptibility to different viral infections ( Table 1). Although in most of the IEI patients, viral agents are not routinely investigated, viral infections have been observed at the same rate in both syndromic and non-syndromic CID patients associated with DNA repair defects. The most frequent monogenic defects reported to be underlying viral infections are RAG deficiency (~45%) and ataxia-telangiectasia (A-T or ATM deficiency, ~15%, Supplementary Figure S1). Of note, DNA repair-deficient patients are at a high risk to develop severe complications after varicella-zoster virus (VZV) (~25%), rubella virus (RuV, ~25%) and cytomegalovirus (CMV, ~20%) infections. Other viral infections affecting a minority of patients include Epstein-Barr virus (EBV), Herpes simplex virus (HSV), rotavirus (RVs), adenovirus (HdAV), influenza virus (IV), metapneumovirus (HMPV), human respiratory syncytial virus (RSV), hepatitis B virus (HBV) and John Cunningham virus (JCV). The most common realm of viral infections in this group of IEI are Duplodnaviria (54%) and Riboviria (42%), and the majority are dsDNA (65%) and ssRNA viruses (26%) [ 107– 120] .

**Table:** **Table 1** Susceptibility to different types of viral infection in patients with inborn errors of immunity (IEI) associated with DNA repair gene defects [ 107– 120]

Virus type	DNA virus	RNA virus
Genome	dsDNA	+ssDNA	dsRNA	+ssRNA	-ssRNA	-ssRNA
Realm	Duplodnaviria	Varidnaviria	Monodnaviria	Riboviria
IEI	Gene defects	VZV	CMV	EBV	HSV	HdAV	JCV	HBV	HMPV	RVs	HRV	RuV	RSV	IV
*Non syndromic CID*	*RAG1*	++	++	+				+						
*RAG2*	++	++	+				+						
*DCLRE1C*											+		
*LIG4*			+								+		+
Syndromic CID	*ATM*											++		
*NBS*											++		
*ZBTB24*		+				+							
*MCM4*	+		+										
*LIG1*	+				+			+	+	+	+	+	
*GINS1*	+	+		+	+				+			+	+

HRV: Rhinovirus.

Among the known DNA repair-related genes in IEIs, *RAG1* and *RAG2* help to create site-specific DSBs, and the RAG complex seals hairpins during antigen receptor recombination. Patients with *RAG1*/ *RAG2* mutations thus have no or low level of V(D)J recombination activity, which leads to a reduction in B- and T-cells numbers and may present as severe CID (SCID) or Omenn syndrome (OS) [ 110, 121] . Different clinical manifestations, including failure to thrive, squamous erythroderma, lymphoproliferation, and severe diarrhea have been described. V(D)J recombination disorders account for 30% of SCID patients, among which approximately 70% are due to *RAG1*/ *RAG2* mutations [122]. In a selected group of patients, hypomorphic *RAG* mutations lead to a ‘leaky’ SCID with a higher rate of DNA lesions [ 123, 124] .

The *PRKDC* gene, encoding the catalytic subunit of a nuclear DNA-PK, drives the broad antiviral response in innate immunity. Human adenovirus 5 and HSV have been shown to block the DNA-PK innate pathway [125]. DNA-PK also plays an important role in adaptive immunity, since it is involved in NHEJ during V(D)J recombination and CSR [126]. Similar to RAG deficiency, one patient previously described with a homozygous missense hypomorphic mutation in *PRKDC* showed both affected DNA lesion sensing and adaptive immune lymphocyte development and later presented with a T-B-SCID phenotype [127].

As mentioned above, ATM is a central element for the DNA damage response. Loss-of-function *ATM* results in A-T, which is a syndromic autosomal recessive disorder. Several studies have indicated that DNA damage due to ATM deficiency leads to an increased production of type I IFN [ 65, 128] . It also affects DSB repair during V(D)J recombination and CSR [ 129, 130] , which leads to restricted T and B cell repertories and reduced switched immunoglobulin level and conversely, increased IgM antibody level [54].

Nibrin, also known as NBS1 or NBN, encodes a protein involved in the DNA-damage kinase activation in the DNA repair process and the MRN complex. NBS is a rare autosomal recessive DNA repair disorder characterized by predisposition to malignancy, ionizing radiation sensitivity and dysregulated cell cycle checkpoint and apoptosis due to mutations in *NBN* [131]. In a study with 35 NBS patients, the Rubella virus was detected by PCR in skin granuloma biopsies from 3 patients (8.5%) [132], suggesting a susceptibility to ssRNA virus in these patients. Due to the role of NBS1/MRN complex in the regulation of NHEJ during V(D)J recombination and CSR, NBS patients may also have altered B cell repertoires and reduced serum IgG and IgA levels [ 133– 135] .

DNMT3B (DNA Methyltransferase 3 Beta) can be recruited to DNA damage sites by proliferating cell nuclear antigen (PCNA) [136]. DNMT3B defects lead to immunodeficiency with centromeric instability and facial anomalies (ICF) syndrome type 1. A previous study showed a high frequency of infection among 23 patients with *DNMT3B* deficiency (ICF1) who had otitis, bronchopneumonia, sepsis, Candida infection or *Pneumocystis jirovecii* infection [137].

Bloom’s syndrome protein, which is involved in HR, may inhibit replication fork progression [138], and mutations in *BLM* lead to Bloom syndrome (BS) which is characterized by growth retardation, genome instability and predisposition to malignancy. The BS patients may also be associated with mild immunodeficiency, with low levels of immunoglobulins [139]. Recurrent bacterial infections can be observed in BS patients, such as upper respiratory and gastrointestinal infections, while viral infections are rare [140].

Taken together, DNA repair defects are in general related to an enhanced susceptibility to viral infections and worse outcomes due to poor cellular immunity and subsequent reduced viral control.

## The SARS-CoV-2 Pandemic and IEI Patients due to DNA Repair Defects

According to the World Health Organization (WHO) SARS-CoV-2 has infected over 510 million people around the world ( https://covid19.who.int/). The case fatality ratio (CFR) has decreased gradually from 10% to 2% in unvaccinated individuals since the very beginning of the pandemic. However, around 10%–20% of those with a confirmed COVID-19 diagnosis become seriously ill and require medical treatment and oxygen therapy [141]. CFR is very low in children and young adults, from 0.002% for those aged 10 to 0.01% for those aged 25 [142]. IEI patients, as a population, have a high risk to develop severe viral disease and may therefore be expected to be more susceptible to develop life-threatening COVID-19. IEI patients may have extra protection due to self-isolation during the COVID-19 pandemic. Although IEI patients show a 1.23-fold higher incidence of SARS-CoV-2 infection, the rate of infection can be much higher than that in the normal population if they are exposed to similar conditions. Moreover, the COVID-19 mortality rate among IEIs is 10-fold higher than in normal individuals [143]. A study with 34 IEI young-adult cases collected from 6 centers indicated that the CFR is 23.5% in young individuals with IEIs [144]. In two other studies, one identified 604 SARS-CoV-2-infected IEI patients with a mortality rate of 8.1%, and another evaluated 94 infected IEI patients, and found a 9.5% overall mortality rate. All studies published till date indicate a higher mortality rate in IEI patients than in the normal population [ 145, 146] , particularly when considering the age composition of the population.

Although the impact of the pandemic is not yet clear in DNA repair-deficient patients, a previous study on a national IEIs cohort showed that patients with DNA repair disorders contributed about 15.8% (3/19) of all IEI cases diagnosed with COVID-19, and showed a mortality rate of 33.3% (1/3) [143]. In another study, where 34 IEIs were randomly recruited from 6 centers, patients with DNA repair disorders constituted the largest part of their cohort (17.6%), including patients with *ATM* ( *n*=4), *NBS* ( *n*=1) and *DNMT3B* ( *n*=1) mutations [144]. However, the clinical manifestations, the final outcomes and therapy data on patients suffering from DNA repair disorders in the current SARS-CoV-2 pandemic are still limited.

**Figure 3:**
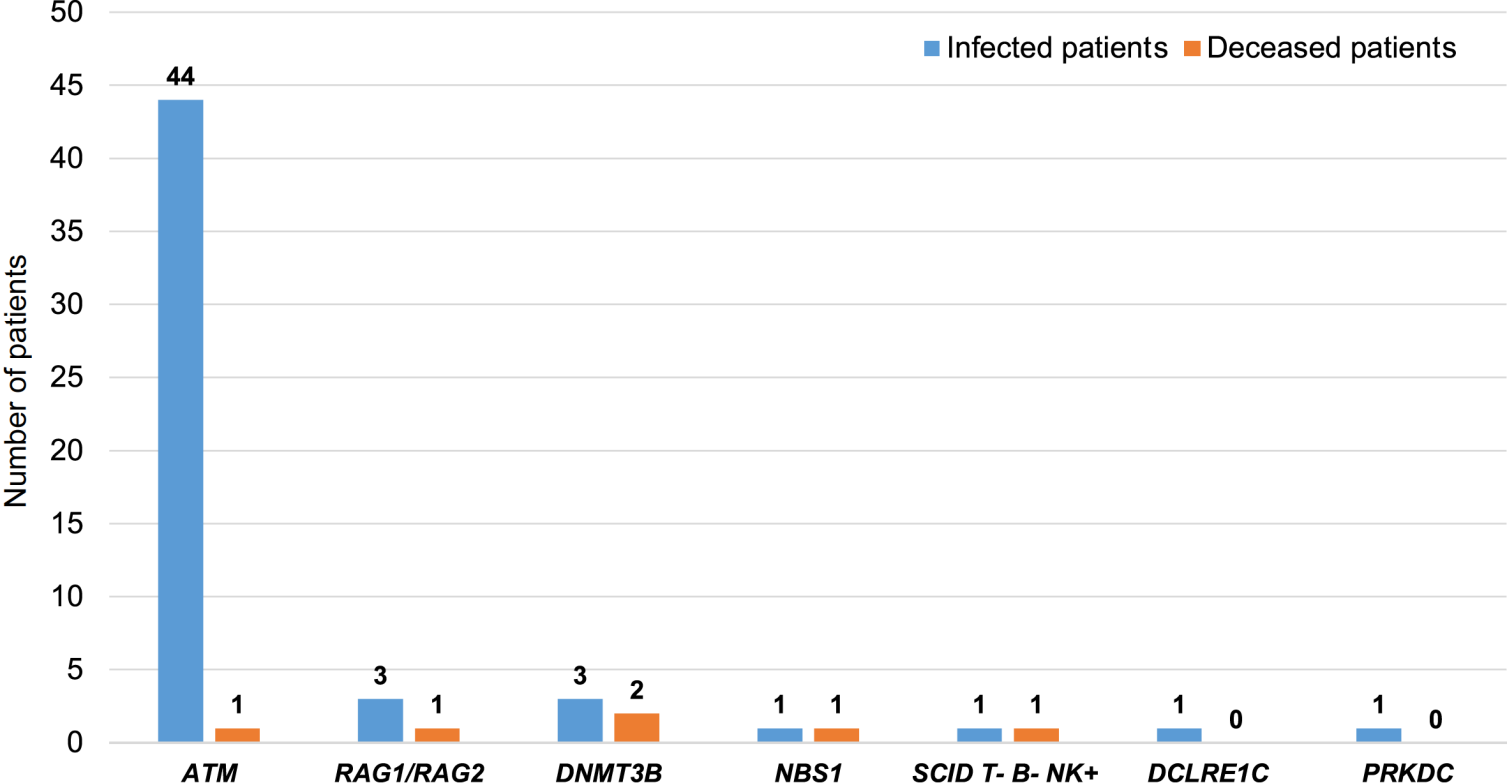
Figure 3 Comparison of the infected patients and deceased patients with IEI associated with DNA repair gene defects (detailed in Supplementary Table S1)

Based on the current reported SARS-CoV-2-infected IEI patients, among the 54 patients with a DNA repair disorder (48% female), 91% of patients had *syndromic CID*s, and 9% patients had non-syndromic CIDs ( Supplementary Table S1). The median age at the time of SARS-CoV-2 infection was 9.3 years. To date, 44 patients had A-T (81%) [ 9, 143, 144, 147– 149] ; 3 patients had RAG deficiency/Omenn syndrome (5%) [ 143, 150, 151] ; 1 patient had DCLRE1C deficiency (2%) [152]; 1 patient had DNA-PKcs deficiency (2%) [145]; 1 patient had NBS (1%) [144]; 3 patients had ICF type 1 (6%) [ 143, 144] and 1 patient had T-B- severe CID without a genetic diagnosis (2%) [143]. The overall mortality rate was 9.2% (5/54) among these patients, which is similar to the mortality rate in the previous 2 IEIs cohorts 8.1% (49/604) and 9.5% (9/94) [ 145, 146] . Of note, a huge difference in the mortality rate exists among DDR subtypes, with 2.27%, 33.33%, and 66.7% in the *ATM*, *RAG1*/ *RAG2* and *DNMT3B* patient groups, respectively ( Figure 3). Life-threatening SARS-CoV-2 infection is surprisingly rare in A-T patients, with only 1 of the 44 patients died (2.27% mortality rate) ( Supplementary Table S1). One possibility is that patients with specific DNA repair disorders, such as A-T patients, develop severe SARS-CoV-2 less frequently, potentially due to an enhanced activation of innate immunity, which is crucial in the early anti-viral response.

## Conclusion

DNA repair disorders are considered to show an intensified innate immune response and reduction of adaptive immunity against SARS-CoV-2 infection. Severe COVID-19 cases are rare in selected DNA repair disorder patients (such as A-T), which may be due to the over-activation of type I IFNs. On the other hand, a higher COVID-19 mortality rate in specific monogenic DNA repair defects (such as RAG1/RAG2, DNMT3B deficiencies *)* exists based on the current observation. Overall, patients with DNA repair defects, especially those at a young age, are at a much higher risk of severe complications, compared to children without pre-existing IEI. It is of particular concern given the note of rapidly increased hospitalization rates among non-vaccinated infants and children due to the circulation of new COVID-19 variant(s). It also highlights the potential benefit of this group of patients from advanced treatments such as passive immunotherapy (hyperimmune immunoglobulin preparations, monoclonal antibodies) [ 153, 154] and specific anti-viral treatment, and the requirement for vaccination prioritization with potent immunization strategy against new variants of concern including Omicron [155].

## Supplementary Data

Supplementary Data is available at *Acta Biochimica et Biphysica Sinica* online.
